# Location, seasonal, and functional characteristics of water holding containers with juvenile and pupal *Aedes aegypti* in Southern Taiwan: A cross-sectional study using hurdle model analyses

**DOI:** 10.1371/journal.pntd.0006882

**Published:** 2018-10-15

**Authors:** Chia-Hsien Lin, Karin Linda Schiøler, Claus Thorn Ekstrøm, Flemming Konradsen

**Affiliations:** 1 Global Health Section, Department of Public Health, University of Copenhagen, Copenhagen, Denmark; 2 Section of Biostatistics, Department of Public Health, University of Copenhagen, Copenhagen, Denmark; University of Heidelberg, GERMANY

## Abstract

**Background:**

*Aedes aegypti* carries several viruses of public health importance, including the dengue virus. Dengue is the most rapidly spreading mosquito-borne viral disease in the world. Prevention and control of dengue mainly rely on mosquito control as there is no antiviral treatment or a WHO-approved vaccine. To reduce the *Ae*. *aegypti* population, studying the characteristics of their habitats is necessary. *Aedes aegypti* prefer breeding in artificial water holding containers in peridomestic or domestic settings. Their juveniles (1^st^ - 4^th^ instar larvae and pupae) have a tendency to cluster in certain types of containers. To inform control strategies, it is important to assess whether the pupae subgroup has a distinct distribution by container type as compared to the overall group of juveniles. The objective of this study was to assess for distinct predictors (location, season, and function) of *Ae*. *aegypti* juveniles and pupae numbers in water holding containers by applying hurdle model analyses.

**Methodology:**

The field component of this study was carried out from November 2013 to July 2015 in Southern Taiwan where annual autochthonous dengue has been reported for decades. Water holding containers with stagnant water were identified in a predefined urban area in Kaohsiung City (KH) and a rural area in Pingtung County. Given that mosquito survey data often include many containers with zero *Ae*. *aegypti*, a negative binomial hurdle model was applied to model the association between location, seasonal and functional characteristics of the water holding containers and the number of *Ae*. *aegypti* in each container.

**Results:**

The results showed that *Ae*. *aegypti* were almost exclusively present in the urban area. In this area, the negative binomial hurdle model predicted significantly more juveniles as well as pupae *Ae*. *aegypti* in water holding containers during the wet season when compared to the dry season. Notably, the model predicted more juveniles in containers located on private property compared to those on government property, irrespective of season. As for pupae, the model predicted higher amounts in indoor containers used for water storage compared to outdoor water storage containers, irrespective of season. However, for the specific category ‘other water receptacle’, higher amounts of pupae were predicted in outdoor compared to indoor in water receptacles, such as flower pot saucers and water catchment buckets.

**Conclusions:**

The difference in predictors for juveniles and the pupae subgroup was identified and it may be of importance to the control strategies of the authorities in KH. At present the authorities focus control activities on all water holding containers found on government property. To improve the ongoing control efforts in KH, the focus of control activities maintained by the KH authorities should be expanded to indoor water storage containers and outdoor water receptacles on both private and government properties to adequately address habitats harboring greater numbers of pupae. In addition, it is proposed to increase community engagement in managing water in all types of water holding containers located on privately owned properties (indoor and outdoor), especially during wet season.

## Author summary

Dengue is considered the most significant mosquito-borne disease, globally. It is transmitted by the mosquito *Aedes aegypti* throughout most affected countries. In order to prevent dengue, most control efforts focus on removing *Ae*. *aegypti* from the environment, as currently there are no anti-viral treatment or WHO approved vaccine. Removing or destroying the breeding sites of *Ae*. *aegypti* is generally recommended. However, this may be difficult as the mosquito breeds in a wide variety of water holding containers readily available in the human environment. The urban areas of Southern Taiwan have experienced annual dengue epidemics for decades in spite of substantial efforts to control *Ae*. *aegypti*. In this study we investigated whether containers harboring the highest number of *Ae*. *aegypti* differ between all active aquatic stages (larvae and pupae) and the final aquatic stage (pupae only). In Kaohsiung City in Southern Taiwan, we found that more *Ae*. *aegypti* were predicted to be in containers on private property, compared with government property, when assessing all aquatic stages. However, for pupae only, more were found in indoor containers used for water storage when compared with outdoor water storage containers. For the specific category ‘other water receptacle’ such as flower pot saucers and water catchment buckets, the model predicted more pupae in outdoor containers compared with indoor containers. We recommend that future control efforts target these types of containers to reduce the risk of dengue transmission in Southern Taiwan.

## Introduction

*Aedes aegypti* is a vector of several viral diseases of public health concern including dengue, which is considered the most important arboviral disease, globally [1, 2]. Dengue presents a formidable public health and economic challenge in most of the affected regions, including Southeast Asia [3, 4]. A tetravalent dengue vaccine was recently marketed, but lack of WHO approval and recent issues pertaining to vaccine safety in the dengue immune, challenge the widespread use of this vaccine [5]. As such, vector control remains the center of dengue prevention options.

During inter-epidemic periods, most *Ae*. *aegypti* control activities aim to limit the transmission potential by reducing the emergence of adult mosquitoes. This is achieved by targeting the aquatic habitats of the immature stages of *Ae*. *aegypti* through source reduction or biological and/or biocidal treatment [2]. Given that pupae is the last of the immature stage and that the pupae mortality rate is low, this aquatic stage is considered a better proxy for adult mosquitoes than eggs and larvae and, therefore, suggested for inclusion in control [1, 6, 7].

The pupal and demographic survey method represents an operational research approach for identifying the types of containers that have the highest rate of adult emergence in a given community [1]. According to Focks et al., most epidemiologically important types of containers can be determined in a given community as pupae per person for each type of container [7–9]. However, as this approach uses the “house” as the survey unit, it may miss the habitats present in other types of properties, such as schools, marketplaces, places of worship and other public spaces.

In Southern Taiwan, autochthonous dengue cases have been reported annually over the past three decades prompting significant top-down government surveillance and control programs in the affected areas [10, 11]. The occurrences of dengue in Southern Taiwan have shown to be associated with general *Stegomyia* indices as well as rainfall and humidity data [12]. Current surveillance activities target the overall group of juveniles (1^st^ - 4^th^ instar larvae and pupae) by inspection of all accessible water holding containers, including public and private areas (interior and exterior). The areas targeted for vector control activities are based on routine assessments of Breteau and Container Indices (BI and CI) across the region. However, using BI or CI for targeting intervention areas and containers has yet to demonstrate a clear impact on the transmission of dengue in Southern Taiwan [10, 11]. Notably, these larval indices do not allow for any potential distinction between containers that harbor low and high numbers of juveniles or for those containers in which the pupae subgroup, may cluster.

The objective of this study was to assess for distinct predictors (location, season, and function) of *Ae*. *aegypti* juveniles and pupae numbers, respectively, in water holding containers found in residential and non-residential areas in different settings of Southern Taiwan, to inform future control activities in Taiwan.

## Materials and methods

### Study area

The survey of juvenile *Aedes* was carried out in the urban setting (>3,500 individuals per km^2^) of Kaohsiung City (KH) and the rural setting (<1,000 individuals per km^2^) of Pingtung County (PT) in Taiwan. Both areas have a long history of recurrent dengue clusters [11]. A case cluster is defined as two new dengue virus confirmed patients either resident or working within a distance of less than 150m apart and their onset of symptoms is within 14 days or less [13].

Kaohsiung City (22°37’N, 120°18’E) is located in southwestern Taiwan. Although the urban setting accounts for only 6.6% of the Kaohsiung City area (194.5 km^2^/2,951.8 km^2^), it holds 67.9% of its population (1.9 million individuals/2.8 million individuals) [14].

In the urban setting of KH, “Li” is the smallest administrative unit, with a mean area of approximately 0.4 km^2^. Piped water coverage is more than 99.2%, yet the tradition of storing water in buckets in the domestic environment remains widespread [15]. Daily household waste removal is carried out by the Environment Protection Bureau, Kaohsiung City Government.

The local authorities, including the Center for Disease Control, Department of Health, Kaohsiung City Government (Kaohsiung CDC), control *Ae*. *aegypti* breeding sites through specially trained vector control personnel, who routinely manage water holding containers throughout the year by direct removal of the containers. For those containers where the water cannot be strained, the authorities apply chemical control methods such as larvicides, including temephos, pyriproxyfen or diflubenzuron or biocontrol methods such as *Bacillus thuringiensis israelensis* (Bti), copepods or larvivorous fish in the containers [13]. These efforts are predominantly limited to government properties. As soon as a dengue cluster occurs, space spraying of pesticide is applied in and around the domestic premises of confirmed cases and their workplaces [13].

Pingtung County (PT) (22°25'N, 120°38'E) has a population of approximately 0.8 million and a population density of 190.1 individuals per km^2^. The village is the smallest administrative unit with a mean area of approximately 5.7 km^2^ in PT. Pingtung County includes some of the most important agricultural areas in Taiwan in terms of irrigated rice fields, aquaculture, and fruit, vegetable and pig farms. It is also one of the least developed counties in Taiwan in terms of infrastructure with less than half of households having access to piped water [15]. The households without access to piped water depend on rainwater collections, groundwater extracted using hand pumps or spring water from the mountains. There are no routine activities aimed at managing of water holding containers for vector control in PT. Vector control interventions, including removal of water holding containers on government property and space spraying of pesticide, are only implemented when case clusters occur, which typically occur on an annual basis during September to December [11].

**Fig 1:**
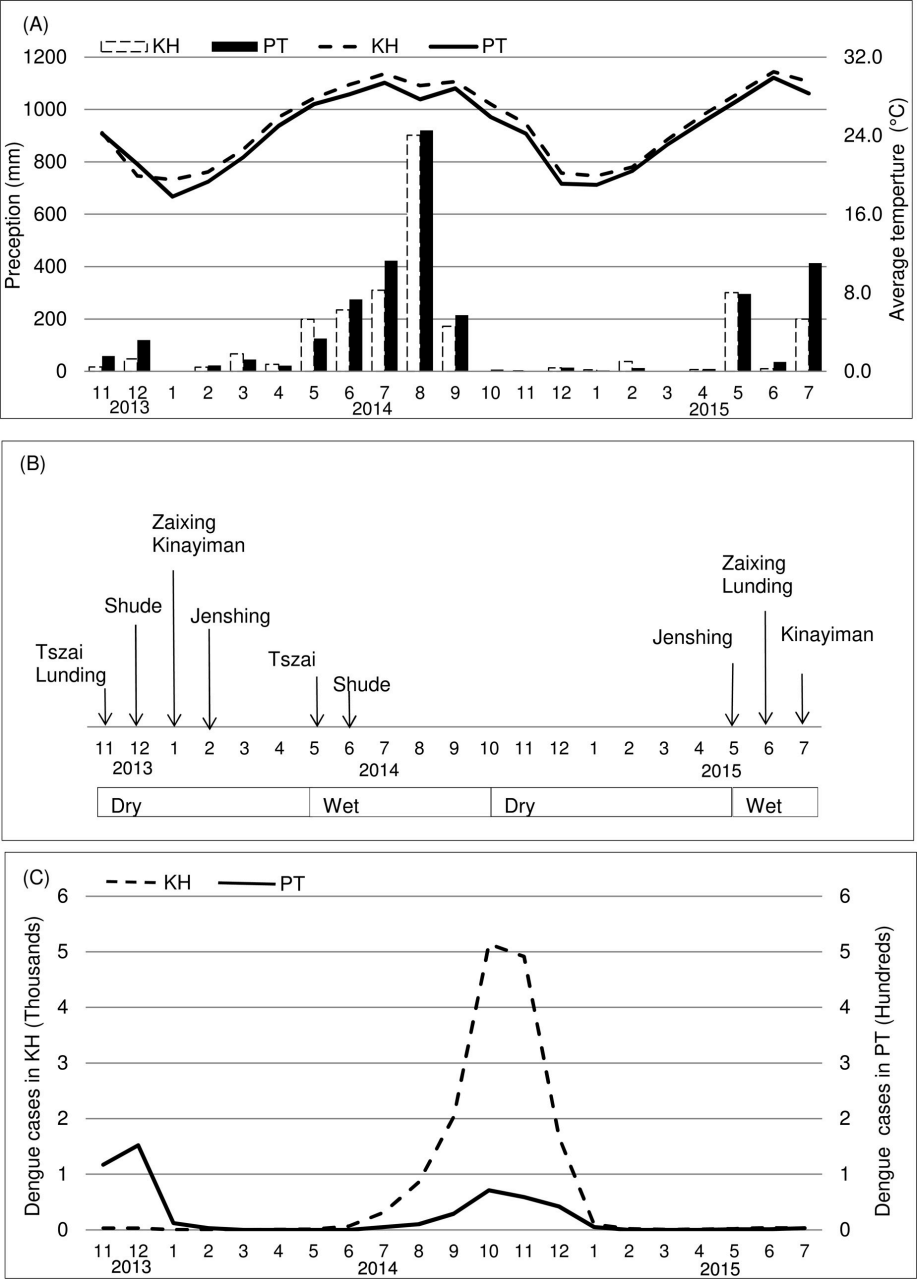
Climatic conditions, timing of mosquito surveys, and confirmed dengue cases in Kaohsiung City (KH) and Pingtung County (PT) Taiwan 2013–15. (A) Monthly precipitation (bars) and average temperature (lines) in KH (dotted) and PT (black). (B) Timeline of mosquito surveys conducted in three urban study sites in KH (Tszai, Shude and Jenshing) and three rural study sites in PT (Kinayiman, Lunding and Zaixing). (C) Epidemic curve of confirmed dengue cases in KH (dotted) and PT (black) reported by Taiwan CDC.

Both Kaohsiung City and Pingtung County have a tropical monsoon climate. The dry season is from October to April and the wet season is from May to September. During the study period (November 2013-July 2015), Kaohsiung City had accumulative precipitations of 241.0 mm during 13 dry months and 2,327.5 mm in the wet season during eight wet months. The lowest temperatures were recorded during December to February with a mean of 20.1°C, while the highest temperatures were observed between June and September with a mean of 29.7°C. Pingtung County followed the same climate pattern as Kaohsiung City (Fig 1A) with distinguishable dry and wet seasons (accumulative precipitations 314.0 and 2,704.0mm, respectively). The average temperature for the coldest months was 19.5°C, as opposed to 28.7°C for the hottest months [16].

### Juvenile *Aedes* survey

**Fig 2:**
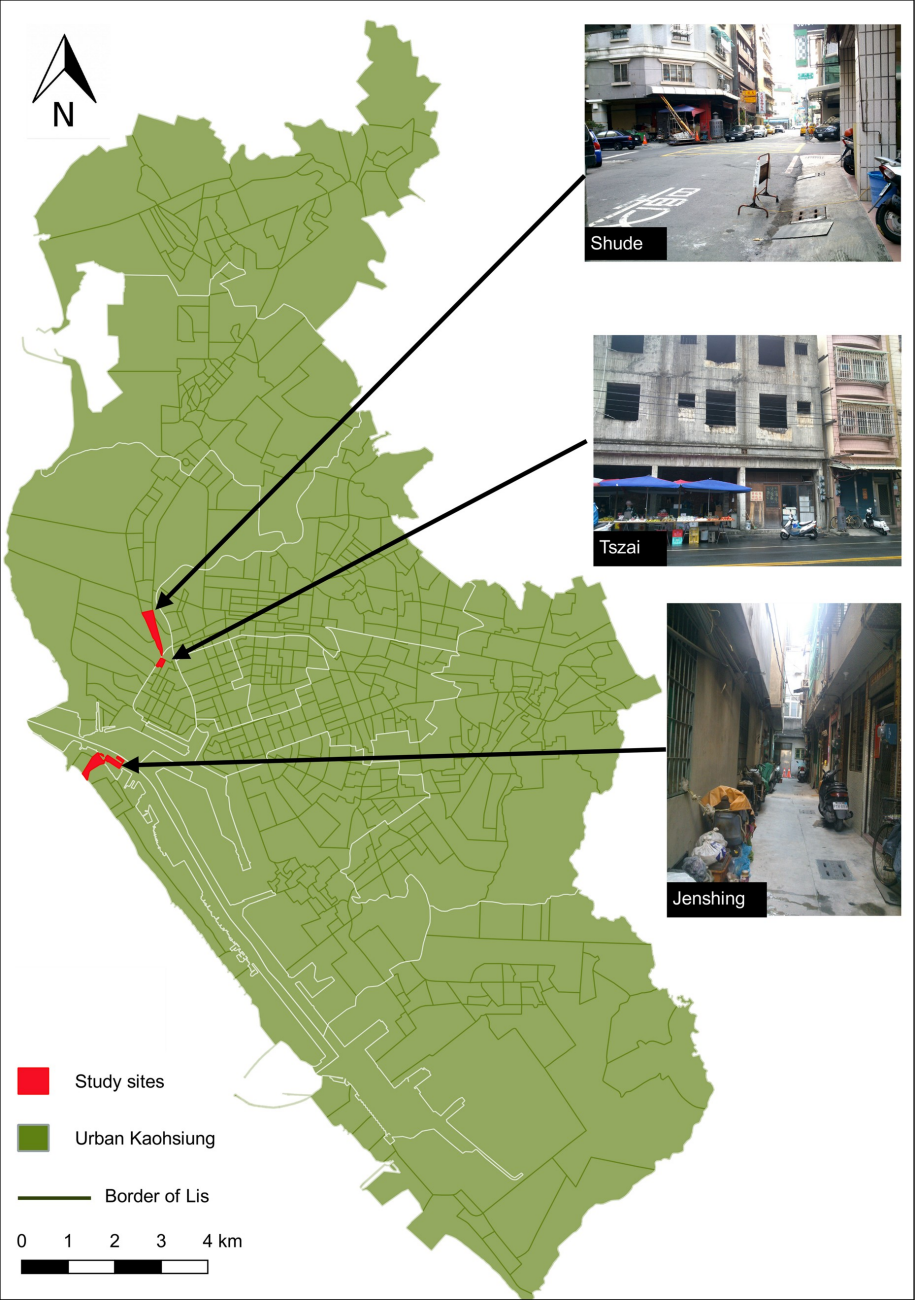
Randomly selected study sites in the urban setting of Kaohsiung City, Taiwan 2013–15. Inserted photos display the typical environment of each study site. Source map: National Land Surveying and Mapping Center [17].

The entomological data collection was conducted at six randomly selected study sites. In the urban setting of KH, three districts were randomly selected from the 12 districts and one Li was randomly chosen from each selected district. The three study sites (Lis) in KH were Tszai, Shude and Jenshing (Fig 2). In the rural setting of PT, three townships were randomly selected from the 28 townships and one village was randomly chosen from each selected township. The three study sites (villages) were Kinayiman, Lunding and Zaixing. Staff resources, availability of equipment, capacity to process samples and time available for the research determined the number of sites included in the study.

One survey in a given study site consisted of two collection periods of four to five consecutive days each. The collection periods were separated by a one-week interval. For each of the six study sites, one survey in the dry season of 2013/2014 (November-February) was completed. In addition, one survey was completed in the wet season of 2014 (May and June) in two study sites (Tszai and Shude), while four surveys (Jenshing, Kinayiman, Lunding and Zaixing) were postponed to the wet season of 2015 (May to July), due to unforeseen circumstances (Fig 1B).

A sample was defined as any water holding container with stagnant water. Sample size (i.e. number of containers) was determined by the set timing for each survey at each study site. Collection activities were undertaken from 9:00 to 11:30 and from 14:00 to 16:30, by a team composed of one researcher and two experienced entomologists from the vector control section of the Kaohsiung CDC. The team surveyed both government and privately owned properties. Private properties were accessed following permission from the owners. For buildings lower than six stories, all accessible households on each floor (including the basement) and space around the buildings were inspected. For buildings higher than six stories, the first five floors were inspected in addition to the basement, attic, rooftop and balcony as well as the space surrounding each building. This is a form of convenience sampling.

From containers with a water volume less than 10L, all water was strained, whereas from larger water holding containers or those difficult to reach, dipping was carried out at the rate of 5 dips per m^2^ of water surface along the edge of the container using a 500ml dipper [18]. All potential *Aedes* were transferred into white bowls for visualization before collection in 25ml glass vials labeled with a sample ID. After daily *Aedes* collection, juvenile *Aedes* were transported from the study site to the laboratory at Kaohsiung CDC. Species identification was completed using standard identification keys by Leopoldo M. Rueda [19]. Each container was recorded by date of sampling as well as the number and stage of juvenile *Aedes* by species. After all containers were identified, each container was characterized by four variables: setting, location, ownership of property and function for human usage. The characterization was to facilitate statistical analysis and later interpretation.

A container was recorded as positive for juveniles if one or more juvenile *Ae*. *aegypti* was found in the given type of container, and were distinguished from those with no juveniles (negative). Similar definitions were applied for the containers positive for pupae, when assessed as a subgroup of juveniles.

### Statistical analysis

We analyzed the container count data with a statistical model that could account for the zeros observed due to non-*Ae*. *aegypti* (negative) containers. A multiple regression model was applied by using the predictors of environmental characteristics of the water holding containers (setting, season, location, ownership, function and their two-way interactions) (Table 1).

**Table 1:** List of variables for water holding containers.

Variables	Category	Definition
Independent		
Setting	Rural	Population density < 1,000 people/km^2^
	Urban	Population density > 3,500 people/km^2^
Season	Dry	October-April
	Wet	May-September
Location	Outdoors	Water holding containers outside of the buildings and unprotected from rainfall by overhanging structure
	Indoors	Water holding containers inside of buildings or protected from rainfall by overhanging structure
Ownership	Government	Buildings or areas owned and managed by the government
	Private	Buildings or areas owned and managed by private persons, companies or organizations
Function	Water storage	Water holding containers for domestic, agricultural, commercial or religious purposes
	Discarded item	Water holding containers with no recognized function for human activities (ground puddles included)
	Other water receptacle	Any water holding containers different to that of water storage and discarded items
Dependent		
The number of juvenile *Ae*. *aegypti* in a given water holding container		
The number of pupae *Ae*. *aegypti* in a given water holding container		

Since the data were collected repeatedly from the same study sites and occasionally from the same containers, we did an initial analysis with a random effect logistic regression model where the association between the presence of juvenile/pupae (positive or negative) and the predictors were analyzed while allowing for correlations (random effects) of study site and container. The random effect was tested by likelihood ratio test (LRT) with chi-square mixture test at a 0.05 significance level.

To handle excess zeros observed in the data, the hurdle model was applied [20–22]. The Poisson hurdle model and the negative binomial hurdle model are both modifications of count models. The modeling process was performed in two stages: i) a logistic regression model was applied to address the positive and negative containers; and ii) variables associated with the number of *Ae*. *aegypti* among positive containers (zero-truncated) were identified by applying Poisson and/or negative binomial distributions, separately [20–22]. The parameters estimated from these two stages were interpreted differently. The exponential of the parameter estimated from the logistic regression model corresponded to the odds ratio for presence of *Ae*. *aegypti*. The exponential of the parameter estimated from the Poisson or negative binomial distribution was the ratio of average number of *Ae*. *aegypti* between two categories. By using a hurdle model, we could estimate the probability of *Ae*. *aegypti* being present, and given *Ae*. *aegypti* being present we would know the relative mean number of *Ae*. *aegypti* in the water holding container [23].

Supervised backward elimination based on chi-square test at a 0.05 significance level was used for determining the predictors for each model. Akaike information criterion (AIC) was used for the selection between Poisson and negative binomial distribution. All models were developed, compared and tested in R version 3.4.1 [24–28].

## Results

### General distribution of water holding containers and juvenile *Aedes aegypti*

During the study period, a total of 897 water holding containers (N) were identified. All identified containers were included in the survey. Notably, using the same sampling effort (by time), nearly equal numbers of containers were identified in the urban (N = 436) and rural (N = 461) settings in both wet (N = 410) and dry (N = 487) seasons. More water holding containers were identified outdoors (N = 683) than indoors (N = 214) and more containers were identified on privately owned properties (N = 603) when government (N = 294) owned properties. As for function, the biggest portion was discarded item (N = 427), followed by water storage (N = 279) and other water receptacle (N = 191) (S1 Table).

Of the 897 water holding containers, 131 (14.6%) and 46 (5.1%) were positive for juvenile and pupae *Ae*. *aegypti*, respectively. More than 95.6% (juveniles 127/131; pupae 44/46) of the positive water holding containers were identified in the urban setting (S1 and S2 Tables). *Aedes aegypti* only co-existed with two mosquito species: *Ae*. *albopictus* in 35 containers and *Culex quinquefasciatus* in six containers. The data for *Aedes albopictus* will be presented in a separate paper.

A total of 4,183 *Ae*. *aegypti* juveniles (S1 Table), including a subgroup of 290 pupae (S2 Table), were collected during the study period, of which the overwhelming majority (juveniles 4,122/4,183, 98.5%; pupae 279/290, 96.2%) were identified in the urban setting of Kaohsiung City (Tables 2 and 3).

**Table 2:** Distribution of containers (N), positive (POS) or negative (NEG) for juvenile *Ae*. *aegypti* (AE) in the urban setting of Kaohsiung City, Taiwan, 2013–2015 (N = 436).

Variable	Category	Number of container			Number of AE
		N (%^a^)	POS (%^b^)	NEG (%^b^)	larvae & pupae (%) ^a^
Season	Wet	217 (50)	81 (37)	136 (63)	2,992 (73)
	Dry	219 (50)	46 (21)	173 (79)	1,130 (27)
Location	Outdoors	303 (69)	86 (28)	217 (72)	2,970 (72)
	Indoors	133 (31)	41 (31)	92 (69)	1,152 (28)
Ownership	Private	219 (50)	75 (34)	144 (66)	2,553 (62)
	Government	217 (50)	52 (24)	165 (76)	1,569 (38)
Function	Water storage	137 (31)	35 (26)	102 (74)	1,230 (30)
	Discarded item	168 (39)	58 (35)	110 (65)	2,134 (52)
	Other water receptacle	131 (30)	34 (26)	97 (74)	758 (18)
Site	Tszai	148 (34)	29 (20)	119 (80)	746 (18)
	Shude	157 (36)	59 (38)	98 (62)	2,186 (53)
	Jenshing	131 (30)	39 (30)	92 (70)	1,190 (29)
Overall		436 (100)	127 (29)	309 (71)	4,122 (100)

^a^ Percentage of identified containers or juveniles for each category within a given variable

^b^ Percentage of AE positive or negative containers within each category

**Table 3:** Distribution of containers (N), positive (POS) or negative (NEG) for pupae *Ae*. *aegypti* (AE) in the urban setting of Kaohsiung City, Taiwan, 2013–2015.

Variable	Category	Number of container			Number of AE
		N (%^a^)	POS (%^b^)	NEG (%^b^)	pupae (%^a^)
Season	Wet	217 (50)	29 (13)	188 (87)	186 (67)
	Dry	219 (50)	15 (7)	204 (93)	93 (33)
Location	Outdoors	303 (69)	28 (9)	275 (91)	174 (62)
	Indoors	133 (31)	16 (12)	117 (88)	105 (38)
Ownership	Private	219 (50)	23 (11)	196 (89)	153 (55)
	Government	217 (50)	21 (10)	196 (90)	126 (45)
Function	Water storage	137 (31)	14 (10)	123 (90)	96 (34)
	Discarded item	168 (39)	19 (11)	149 (89)	81 (29)
	Other water receptacle	131 (30)	11 (8)	120 (92)	102 (37)
Site	Tszai	148 (34)	10 (7)	138 (93)	59 (21)
	Shude	157 (36)	16 (10)	141 (90)	116 (42)
	Jenshing	131 (30)	18 (14)	113 (86)	104 (37)
Overall		436 (100)	44 (10)	392 (90)	279 (100)

^a^ Percentage of identified containers or pupae for each category within a given variable

^b^ Percentage of AE positive or negative containers within each category

Given that the vast majority of *Ae*. *aegypti* juveniles and pupae as well as *Ae*. *aegypti* positive containers were identified in the urban setting, all subsequent analyses presented in this paper were completed using the data from Kaohsiung City only (N = 436).

### Distribution of water holding containers and juvenile *Aedes aegypti* in Kaohsiung City

We identified 436 containers in the three randomly selected study sites: Tszai (N = 148), Shude (N = 157) and Jenshing (N = 131) located in the urban setting of KH. From the 436 containers, 25 were surveyed twice. As in the case of all containers, those identified in the urban setting of KH were almost equally distributed between seasons (wet = 217, dry = 219). The number of outdoor containers (N = 303) were notably larger than that of indoor containers (N = 133) while there was near equal distribution in terms of private (N = 219) vs. government (N = 217) ownership as well as for function in terms of water storage (N = 137), discarded item (N = 168) and other receptacle (N = 131) (Table 2).

Of the 436 water holding containers identified in KH, 127 (29.1%) were positive for juvenile *Ae*. *aegypti*, harboring a total of 4,122 juveniles (Table 2). A total of 279 pupae were identified in 44 (10.1%) containers (Table 3).

### Characteristics of the water holding containers associated with juvenile *Aedes aegypti* in Kaohsiung City

According to the applied likelihood ratio test, there was neither statistically significant random effect of container (p = 0.810) nor of study site (p = 1.000).

Due to a lower AIC, applying the negative binomial hurdle model to assess the association (AIC = 1594.5) was seen as superior in comparison to the Poisson hurdle model (AIC = 7276.9). The negative binomial hurdle model showed that season (p<0.001) and ownership (p = 0.053) were associated with the number of juveniles. Note that ownership was marginally significant as its p-value was close to the cutoff level (0.05).

Logistic regression showed that containers identified in the wet season had 2.2 times (95% CI = 1.5–3.4) the odds of harboring juvenile *Ae*. *aegypti* compared with containers in the dry season. Also, those sampled on private properties had 1.7 times greater odds (95% CI = 1.1–2.5) of being infested compared with containers on government properties (Table 4, S3 Table).

**Table 4:** Negative binomial hurdle model for juvenile *Ae*. *aegypti* in Kaohsiung City, Taiwan (N = 436).

		Est	SE	Exp(Est)	95% CI**
Negative binomial model*					
	Season (wet vs. dry)	0.5	0.3	1.7	0.9–3.2
	Ownership (private vs. government)	0.2	0.3	1.2	0.7–2.4
Logistic regression model					
	Season (wet vs. dry)	0.8	0.2	2.2	1.5–3.4
	Ownership (private vs. government)	0.5	0.2	1.7	1.1–2.5

* Zero-truncated

** 95% CI referring to Exp(Est)

Conditional on logistic process, however, zero-truncated negative binomial model showed that the average number of juveniles in each juvenile positive container was not significantly associated with season (1.7, 95% CI = 0.9–3.2) or ownership (1.2, 95% CI = 0.7–2.4) (Table 4).

### Characteristics of the water holding containers associated with pupae *Aedes aegypti* in Kaohsiung City

According to LRT, the model found that there were neither statistically significant a random effect of container (p = 0.733) nor of study site (p = 1.000).

For the pupae stage, the negative binomial hurdle model (AIC = 521.9) was observed as substantially better than the Poisson hurdle model (AIC = 626.8). Here, season (p = 0.036) as well as an interaction between location and function (p = 0.009) were identified as predictors.

Logistic regression showed that the odds of harboring pupae *Ae*. *aegypti* were 2.1 times (95% CI = 1.1–4.0) greater for containers sampled in the wet season when compared to the dry season (Table 5, S3 Table).

**Table 5:** Negative binomial hurdle model for pupae *Ae*. *aegypti* in Kaohsiung City, Taiwan (N = 436).

		Est	SE	Exp(Est)	95% CI**
Negative binomial model*					
	Season (wet vs. dry)	-0.3	0.5	0.7	0.3–2.0
	Location x Function				
	Out vs. In (water storage)	-1.6	0.8	0.2	0.1–0.9
	Out vs. In (other receptacle)	2.2	0.9	8.8	1.4–56.3
	Out vs. In (discarded)	0.9	1.1	2.3	0.3–21.5
Logistic regression model					
	Season (wet vs. dry)	0.7	0.3	2.1	1.1–4.0

* Zero-truncated

** 95% CI referring to Exp(Est)

For each pupae positive container, the average number of pupae was not significantly associated with season (0.7, 95% CI = 0.3–2.0). However, the average number of pupae per positive container was 5.0 times (95% CI = 1.1–23.7) as high for indoor as outdoor containers categorized as water storage containers (S4 Table). Nevertheless, the average number of pupae per positive container was 8.8 times (95% CI = 1.4–56.3) higher for outdoor as compared with indoor containers categorized as ‘other water receptacle’. Meanwhile, the average number of pupae per positive container was not significantly different between outdoor and indoor containers categorized as ‘discarded item’ (2.3, 95% CI = 0.3–21.5) (Table 5, S3 Table).

## Discussion

We found *Ae*. *aegypti* to be almost exclusively present in the urban setting with notable differences between the characteristics of water holding containers harboring the highest number of juveniles and those harboring the highest number of pupae. The hurdle model predicted that water holding containers identified during the wet season had significantly more juveniles and pupae, when compared to containers identified in the dry season. Irrespective of season, containers located on private properties harbored significantly more juveniles compared with those on government properties, while water storage containers used indoors for domestic, commercial or religious purposes had significantly more pupae than those identified outdoors. Other water receptacles, identified outdoors, such as saucers underneath flower pots and buckets for catching leaking water, harbored significantly more pupae than similar kinds of containers identified indoors.

### Distribution of *Aedes aegypti* in rural settings

In the late 1980s, *Ae*. *aegypti* was identified in almost 50% (13 out of 28) rural townships in Pingtung County [29]. However, during 2004–2008, Tuan et al. conducted the surveys in all townships in PT, but *Ae*. *aegypti* juveniles were only found in three rural townships (Ligang, Chunri and Sandimen) [30]. In our study, all *Ae*. *aegypti* juveniles were identified in one rural township (Chunri). The observed decrease in *Ae*. *aegypti* abundance was also reported in 23 villages in Haikou city, South China [31]. The surveys conducted by Su et al. showed that out of 23 villages, 22, 17 and 2 *Ae*. *aegypti* positive villages were observed in 1983, 1993–1994 and 2002, respectively [31].

There is no clear explanation for the disappearance of *Ae*. *aegypti* from rural PT given that control activities in this county have been largely limited to the periods of occurrences of case clusters, unlike the perennial control activities maintained in urban Kaohsiung. The observed decrease in *Ae*. *aegypti* abundance in the rural setting maybe the result of mating interference with male *Ae*. *albopictus* or resource competition with *Ae*. *albopictus* larvae [32, 33].

### *Aedes aegypti* and water storage practices in urban Kaohsiung

Poor access to piped water supply is often cited as one of the main reasons for high numbers of water storage containers suitable for *Ae*. *aegypti* breeding [34, 35]. However, even though water supply coverage in this study was almost 100% in the urban setting and only 50% in the rural area [15], we found almost the same proportion of water storage containers in urban (N = 137) and rural (N = 142) areas (S5 Table). Based on field observations, water storage containers in the urban setting were maintained for religious and commercial purposes. However, in the rural area, water was stored for domestic and agricultural usages.

In our study, indoor water storage containers harbored significantly more *Ae*. *aegypti* pupae, when compared to outdoor water storage containers in the urban setting. A pupal/demographic survey in urban Thailand found that during the dry season more than 70% of all pupae were harbored in indoor containers, toilet tanks, flower pots and plastic buckets [7]. However, it was not clearly defined if the flower pots and plastic buckets were found indoors or outdoors.

### *Aedes aegypti* in the wet and dry seasons

Our study indicated that water holding containers identified in the wet season harbored significantly more juveniles when compared to similar containers during the dry season and the same pattern was observed for pupae. This seasonal pattern is in agreement with the previous findings from Taiwan as well as Singapore, China and South and Southeast Asia [10, 30, 36–40].

For *Ae*. *aegypti* pupae, the evidence is inconclusive as to whether containers harboring the highest number of *Ae*. *aegypti* pupae differ between wet and dry seasons. In our study, the model showed that the interaction between season and function was not a predictor for *Ae*. *aegypti* pupae (Table 5). This implies that type (function) of containers harboring the highest number of pupae in the wet season were the same as during the dry season. Our results are in line with a study conducted by Tsuzuki et al. in Vietnam [37]. Tsuzuki’s study showed, by applying convenience sampling, that in one study area the same highly productive containers (harboring >70% of all pupae) were identified in the dry and wet seasons including wells, flower vases and concrete toilet basins. However, from another study area in Vietnam reported in the same publication, different types of highly productive containers were identified in dry and wet seasons [37]. Differences in types of productive containers between seasons were also found in a study conducted by Wai et al. [38]. Wai undertook pupae surveys to identify the types (function) of productive containers (>70% of all pupae) in the six South and Southeast Asia countries [38].

### Containers on privately and government owned properties

Our results indicated that on average there were more juvenile *Ae*. *aegypti* present in the containers located on private properties than in containers located on government properties. The ownership of property was a predictor of juveniles only. However, the p-value (0.053) was marginally significant, so the interpretation of ownership should be taken with caution and further studies would be needed to draw strong conclusions. Future research is needed to investigate if differences in *Ae*. *aegypti* abundance between water holding containers located on private and government properties can be explained by the type of property (commercial, residential, recreational) and where present the type of house (abandoned, residential or commercial) and structure of the house (high-rise/low-rise or with/without basement). The difference may also be explained by the biological and chemical elements in the water between the two properties.

According to our definition (Table 1), water holding containers on private property not only include the containers in private residential areas but also those on privately owned but shared space such as flooded basements, the rooftop and common garden areas. Flooded basements were identified as one of the important *Ae*. *aegypti* breeding sites in Hwang’s study in KH [41]. Hwang et al. surveyed 7,773 dwellings and found 552 had flooded basements. Among the 552 basements, 130 were positive for *Ae*. *aegypti* juveniles [41]. In addition, water tanks on the rooftop or tree holes or standing ornamental fountains in common garden areas may have also acted as *Ae*. *aegypti* breeding sites. Routine vector surveillances conducted by KH authorities in 2017 showed that among 126,260 water holding containers identified in KH, 8,546 containers were positive for *Aedes* juveniles and 9.2% (789 out of 8,546,) were water tanks on the rooftop [42].

In our study, it was not possible to identify the specific type of water holding containers harboring the most *Aedes* on private property. Surveillance results from a similar setting in Singapore indicated that the most frequent domestic *Aedes* habitats included plants, flower pots and ornamental containers [43].

### Implications of study findings for vector control in Kaohsiung City

#### Public communication

The public will not be in a position to differentiate between the different aquatic stages of *Ae*. *aegypti* and health communications therefore need to stress the importance of managing all water holding containers on private properties, irrespective of location (indoor/outdoor) and function, with special emphasis given to the management of containers during the wet season.

Since 2002, Kaohsiung City government has launched a range of public campaigns, aimed at dissemination information on dengue through social media, radio, television and posters. In addition, the authorities encourage the community to engage in the removal or management of water holding containers in and around their houses to control *Ae*. *aegypti* [44]. Pat et al evaluated effects of campaign activities in 2002 and found that the ovitrap index dropped from 67% to 39% three months after introducing a one-week community-based campaign in Kaohsiung [44]. Our study documented a greater number of *Ae*. *aegypti* in containers on private property compared with government property, supporting interventions aimed at targeting owners of a private property in future campaigns. From field observations made during the implementation of this study we noticed that many containers had *Aedes* eggs on the inner walls of the containers. It seemed like people just emptied or refilled the containers without scrubbing the insides of the vessels. This may reflect a low awareness among the inhabitants on how best to manage containers to reduce *Aedes* propagation. Further investigations and new approaches are needed to design interventions to increase the engagement of the public in vector control in the domestic environment. It is likely that behavior change or general educational campaigns have to be tailored to different age groups, especially high risk age groups for dengue infections, with suitable methods, such as door-to-door visits, school dramas, a short video clip before a movie or targeted social media promotions [45–47].

For the shared but privately owned space such as basements, enclosed yards or rooftops, we suggest that the authorities guide the community members to identify the potential *Aedes* habitats in the common spaces and encourage the inhabitants to take responsibility to manage the environment.

#### Dengue vector control personnel

Although the local authorities in KH have put a lot of effort into the removal and management of water holding containers on government property and have performed emergency insecticide spraying as soon as a dengue cluster occurs, the case clusters have occurred over the past decades showing that *Aedes* populations are not sufficiently suppressed to prevent dengue transmissions. As the pupal stage is considered a proxy for the adult stage [1], for the government staff to prioritize resources, we suggest that the authorities focus on the controls of pupae. According to the findings of this study, this would imply a focus on managing water holding containers identified during the wet season. In addition, the focus also included containers found outdoors classified as ‘other water receptacle’ such as flower pot saucers and rain gutters. Finally, control will have to include containers used for water storage indoors.

Our findings clearly support the current surveillance and control focus on water holding containers in the urban environment during the wet season. However, the routine control of water holding containers on government property, located in parks and long roads, may still overlook less accessible but important, outdoor pupae habitats, including extensively used covers and tarpaulins to protect against sun/rain, flower pot saucers and rain gutters. To control pupae in those containers, Aquatain AMF (polydimethylsiloxane, a monomolecular film) or larvicidal oil can be applied [48].

Importantly, control efforts should be expanded to the indoor environment of both government and private properties with respect to water storage containers, specifically. From field observations made as part of this study, the public seemed fatigued by the repeated surveillances from different units (Taiwan CDC, Kaohsiung CDC, Public Health centers of Kaohsiung City and the Environment Protection Bureau of Kaohsiung City). Improved coordination between the authorities may improve the success rate to access indoor containers on private properties. In our survey, indoor water storage containers included vases, pet drinking containers and buckets in the bathroom for multiple usages. Although, not covered by the objectives of this study it seems unlikely that communities in our study area would accept the application of, for example, Bti to this type of indoor domestic containers. Managing such indoor water storage containers on private properties would mainly rely on the owners themselves. Further studies will be needed to identify the best way forward to ensure such behavioral change.

It is likely that a targeted control of *Ae*. *aegypti* control efforts would have a positive spill-over effect on other mosquito species in KH, as we identified co-existence of *Ae*. *aegypti* with *Ae*. *albopictus* and *Culex* across all types of targeted containers.

### Excess zeros

From an ecological perspective, excess zeros (negative containers) may be of two types: true zeros and false zeros [22, 23]. In this study, the true zeros would include water holding containers where no *Ae*. *aegypti* were identified because the environment of the container was not suitable for *Ae*. *aegypti*. However, false zeros may result from the particular study design and sampling methods applied. For the 16 water holding containers with a large water volume identified or being difficult to reach in this study, *Ae*. *aegypti* may have existed but the dipping techniques applied may not have captured *Ae*. *aegypti* if present in low numbers (S1 Data). Furthermore, newly hatched larvae may be particularly difficult to detect increasing the risk of generating false zeros. Finally, false zeros may have resulted from misidentification of species or other reasons.

Two different models are generally used to handle excess zeros in data: a zero-inflated model and a hurdle model [22, 23]. An analysis applying a zero-inflated model is recommended if it is not known whether the observed zeros are true or false zeros. However, in this study it was assumed that all zeros were true zeros, i.e. all zeros were due to an unfavorable environment for harboring *Ae*. *aegypti*, hence a hurdle model was applied. This assumption was based on urban Kaohsiung having ideal temperature for the propagation of *Ae*. *aegypti* all year. The temperature in urban KH is between 20–30°C and this is suitable for *Ae*. *aegypti* development from eggs to adults [1, 16, 49, 50]. In addition, previous studies and from this study *Ae*. *aegypti* were found to exploit a great diversity of materials and functions of water holding containers as habitats (S6 Table) [30, 41, 42]. Finally, sampling events took place four times at each study site and careful sampling was done to increase the likelihood of identifying *Ae*. *aegypti* in suitable water holding containers. From a statistical perspective, the key assumption behind the selection of a hurdle model is that all water holding containers with suitable environments for *Ae*. *aegypti* harbor *Ae*. *aegypti*.

Even though a zero-inflated model and a hurdle model dealt with zeros with different approaches, at the stage of analysis we did apply the hurdle model analyses and zero-inflated model analyses with Poisson and negative binomial distributions to compare results (S7 Table) [22, 23]. Applying the negative binomial hurdle model and the negative binomial zero-inflated model, the same predictors and AICs for juveniles and pupae were identified.

### Strengths and limitation

All identified containers were characterized after the collection with photographs and written descriptions. They were classified by the same researcher to ensure consistency in classification. In addition, supervised sampling in the field was conducted by experienced entomologists.

Convenience sampling was conducted in each study site. Convenience sampling is useful in exploratory research and this method is relatively inexpensive and easy to conduct. However, convenience sampling is a nonprobability sampling method, which means in this survey the samples may not represent the overall population (all containers in the study sites).

If the time and the resources were available, the intensity and frequency of sampling could have been increased. An increase in collection frequency may have resulted in different profiles of containers due to intra-seasonal variations. In addition, the likelihood of identifying the transient water holding containers would increase.

Similar to the pupal/demographic survey approach, our approach was also more labor intensive than the traditional *Aedes* indices [1]. In addition, our approach and pupal/demographic survey cannot identify the unapparent habitats. When sampling large containers, it is difficult to get the actual *Aedes* numbers from both approaches [7].

In the survey, the data on the number of *Aedes* collected in each container were based on two different methods: all *Ae*. *aegypti* in a small container vs. a portion of *Ae*. *aegypti* by dipping in a large container. Combining the numbers of *Ae*. *aegypti* from these two collection methods could be a limitation. However, dipping techniques were only applied for 3.7% (16 out of 436) water holding containers. In these 16 containers, only three were positive for juvenile *Ae*. *aegypti*, harboring a total of six juveniles (S1 Data).

The number of households where we could not gain access due to occupants being absent was not recorded and may have introduced a bias as they might represent a different container profile. Only a small portion (< 5.0%) of households refused the research team entry to their property.

## Supporting information

S1 Table Distribution of containers (N), positive (POS) or negative (NEG) for juvenile *Ae*. *aegypti* (AE) in the urban setting of Kaohsiung City and rural Pingtung County, Taiwan, 2013–2015. (DOCX)

S2 Table Distribution of containers (N), positive (POS) or negative (NEG) for pupal *Ae*. *aegypti* (AE) in the urban setting of Kaohsiung City and rural Pingtung County, Taiwan, 2013–2015. (DOCX)

S3 Table Candidate models for juvenile and pupae *Ae*. *aegypti*. (DOCX)

S4 Table Negative binomial hurdle model for pupae *Ae*. *aegypti* with “outdoors” as the reference group. (DOCX)

S5 Table Distribution of water holding containers between setting and function (setting x function). (DOCX)

S6 Table The number of containers positive for juvenile *Ae*. *aegypti* by characteristics. (DOCX)

S7 Table Zero-inflated model vs. zero-truncated model. (DOCX)

S1 Data Data of all identified water holding containers (N = 897) in urban Kaohsiung City and rural Pingtung County, Taiwan. (XLSX)
