# Supplementary material for: Location, seasonal, and functional characteristics of water holding containers with juvenile and pupal Aedes aegypti in Southern Taiwan: A cross-sectional study using hurdle model analyses
Source: PLoS Negl Trop Dis. 2018 Oct 15;12(10):e0006882. doi: 10.1371/journal.pntd.0006882 (PMC6201951; doi:10.1371/journal.pntd.0006882)
Supplement: S1 Table — (DOCX) [file pntd.0006882.s001.docx]

| **S1 Table.** Distribution of containers (N), positive (POS) or negative (NEG) for juvenile *Ae. aegypti* (AE) in the urban setting of Kaohsiung City and rural Pingtung County, Taiwan, 2013-2015 (N=897). | | | | | | |
| --- | --- | --- | --- | --- | --- | --- |
| Variable | Category | Number of container | | |  | Number of AE |
|  |  | N (%^a^) | POS (%^b^) | NEG (%^b^) |  | (larvae + pupae) |
| Setting | Urban | 436 (49) | 127 (29) | 309 (71) |  | 4,122 |
|  | Rural | 461 (51) | 4 (1) | 457 (99) |  | 61 |
| Season | Wet | 410 (46) | 83 (20) | 327 (80) |  | 3,037 |
|  | Dry | 487 (54) | 48 (10) | 439 (90) |  | 1,146 |
| Location | Outdoors | 683 (76) | 89 (13) | 594 (87) |  | 3,019 |
|  | Indoors | 214 (24) | 42 (20) | 172 (80) |  | 1,164 |
| Ownership | Private | 603 (67) | 79 (13) | 524 (87) |  | 2,614 |
|  | Government | 294 (33) | 52 (18) | 242 (82) |  | 1,569 |
| Function | Water storage | 279 (31) | 36 (13) | 243 (87) |  | 1,234 |
|  | Discarded item | 427 (48) | 60 (14) | 367 (86) |  | 2,179 |
|  | Other water receptacle | 191 (21) | 35 (18) | 156 (82) |  | 770 |
| Overall |  | 897 (100) | 131 (15) | 766 (85) |  | 4,183 |
| ^a^ Percentage of identified containers for each category within a given variable | | | | | | |
| ^b^ Percentage of AE positive or negative containers within each category | | | | | | |
