# Supplementary material for: Location, seasonal, and functional characteristics of water holding containers with juvenile and pupal Aedes aegypti in Southern Taiwan: A cross-sectional study using hurdle model analyses
Source: PLoS Negl Trop Dis. 2018 Oct 15;12(10):e0006882. doi: 10.1371/journal.pntd.0006882 (PMC6201951; doi:10.1371/journal.pntd.0006882)
Supplement: S2 Table — (DOCX) [file pntd.0006882.s002.docx]

| **S2 Table.** Distribution of containers (N), positive (POS) or negative (NEG) for pupal *Ae. aegypti* (AE) in the urban setting of Kaohsiung City and rural Pingtung County, Taiwan, 2013-2015 (N=897). | | | | | | |
| --- | --- | --- | --- | --- | --- | --- |
| Variable | Category | Number of container | | |  | Number of AE |
|  |  | N (%^a^) | POS (%^b^) | NEG (%^b^) |  | (pupae) |
| Setting | Urban | 436 (49) | 44 (10) | 392 (90) |  | 279 |
|  | Rural | 461 (51) | 2 (0) | 459 (100) |  | 11 |
| Season | Wet | 410 (46) | 31 (8) | 379 (92) |  | 197 |
|  | Dry | 487 (54) | 15 (3) | 472 (97) |  | 93 |
| Location | Outdoors | 683 (76) | 30 (4) | 653 (96) |  | 185 |
|  | Indoors | 214 (24) | 16 (7) | 198 (93) |  | 105 |
| Ownership | Private | 603 (67) | 25 (4) | 578 (96) |  | 164 |
|  | Government | 294 (33) | 21 (7) | 273 (93) |  | 126 |
| Function | Water storage | 279 (31) | 14 (5) | 265 (95) |  | 96 |
|  | Discarded item | 427 (48) | 21 (5) | 406 (95) |  | 92 |
|  | Other water receptacle | 191 (21) | 11 (6) | 180 (94) |  | 102 |
| Overall |  | 897 (100) | 46 (5) | 851 (95) |  | 290 |
| ^a^ Percentage of identified containers for each category within a given variable | | | | | | |
| ^b^ Percentage of AE positive or negative containers within each category | | | | | | |
