# Supplementary material for: Location, seasonal, and functional characteristics of water holding containers with juvenile and pupal Aedes aegypti in Southern Taiwan: A cross-sectional study using hurdle model analyses
Source: PLoS Negl Trop Dis. 2018 Oct 15;12(10):e0006882. doi: 10.1371/journal.pntd.0006882 (PMC6201951; doi:10.1371/journal.pntd.0006882)
Supplement: S3 Table — (DOCX) [file pntd.0006882.s003.docx]

| **S3 Table.** Candidate models for juvenile and pupae *Ae. aegypti*. | | | | |
| --- | --- | --- | --- | --- |
|  | Model | AIC | Zero-truncated NB | Logistic regression model |
| Juvenile | M1.NB | 1594.501 | season + ownership | season + ownership |
|  | M2.NB | 1597.963 | season + ownership | season |
|  | M3.NB | 1606.559 | season + ownership | ownership |
|  | M4.NB | 1610.167 | season + ownership | 1 |
|  |  |  | Zero-truncated Poisson | Logistic regression model |
|  | M1.P | 7283.870 | season + ownership + location + function + season: function + ownership: function + location: function | season + ownership + location + function + season: function + ownership: function + location: function |
|  | M2.P | 7276.944 | season + ownership + location + function + season: function + ownership: function + location: function | season |
|  | M5.P | 7289.147 | season + ownership + location + function + season: function + ownership: function + location: function | 1 |
|  |  |  | Zero-truncated NB | Logistic regression model |
| Pupae | M1.NB | 524.1500 | season + location + function + location: function | season + location + function + location: function |
|  | M2.NB | 521.8926 | season + location + function + location: function | season |
|  | M3.NB | 528.4336 | season + location + function + location: function | location+ function+ location: function |
|  | M4.NB | 525.0696 | season + location + function + location: function | 1 |
|  |  |  | Zero-truncated Poisson | Logistic regression model |
|  | M1.P | 632.9670 | season + ownership + location + function + season: function + ownership: location + location: function | season + ownership + location + function + season: function + ownership: location + location: function |
|  | M2.P | 626.7634 | season + ownership + location + function + season: function + ownership: location + location: function | season |
|  | M3.P | 633.2354 | season + ownership + location + function + season: function + ownership: location + location: function | function |
|  | M4.P | 633.3044 | season + ownership + location + function + season: function + ownership: location + location: function | location + function + location: function |
|  | M5.P | 629.0209 | season + ownership + location + function + season: function + ownership: location + location: function | season + location + function + location: function |
|  | M6.P | 629.9404 | season + ownership + location + function + season: function + ownership: location + location: function | 1 |
