# Supplementary material for: Location, seasonal, and functional characteristics of water holding containers with juvenile and pupal Aedes aegypti in Southern Taiwan: A cross-sectional study using hurdle model analyses
Source: PLoS Negl Trop Dis. 2018 Oct 15;12(10):e0006882. doi: 10.1371/journal.pntd.0006882 (PMC6201951; doi:10.1371/journal.pntd.0006882)
Supplement: S4 Table — (DOCX) [file pntd.0006882.s004.docx]

| **S4 Table.** Negative binomial hurdle model for pupae *Ae. aegypti* in Kaohsiung City, Taiwan (N=436). | | | | | |
| --- | --- | --- | --- | --- | --- |
|  |  | Est | SE | Exp(Est) | 95% CI** |
| Negative binomial model* | | |  |  |  |
|  | Season (wet vs. dry) | -0.3 | 0.5 | 0.7 | 0.3-2.0 |
|  | Location x Function |  |  |  |  |
|  | **In vs. Out** (water storage) | 1.6 | 0.8 | 5.0 | 1.1-23.7 |
|  | **In vs. Out** (other receptacle) | -2.2 | 0.9 | 0.1 | 0.0-0.7 |
|  | **In vs. Out** (discarded) | -0.9 | 1.1 | 0.4 | 0.0-3.9 |
| Logistic regression model | | |  |  |  |
|  | Season (wet vs. dry) | 0.7 | 0.3 | 2.1 | 1.1-4.0 |
| * Zero-truncated | |  |  |  |  |
| ** ** 95% CI referring to Exp(Est) | |  |  |  |  |
