# Supplementary material for: Location, seasonal, and functional characteristics of water holding containers with juvenile and pupal Aedes aegypti in Southern Taiwan: A cross-sectional study using hurdle model analyses
Source: PLoS Negl Trop Dis. 2018 Oct 15;12(10):e0006882. doi: 10.1371/journal.pntd.0006882 (PMC6201951; doi:10.1371/journal.pntd.0006882)
Supplement: S5 Table — (DOCX) [file pntd.0006882.s005.docx]

| **S5 Table.** The number of water holding containers between setting and function. | | | |
| --- | --- | --- | --- |
| Setting / Function | Water storage | Discarded item | Other water receptacle |
| Urban | 137 | 168 | 131 |
| Rural | 142 | 259 | 60 |
