# Supplementary material for: Location, seasonal, and functional characteristics of water holding containers with juvenile and pupal Aedes aegypti in Southern Taiwan: A cross-sectional study using hurdle model analyses
Source: PLoS Negl Trop Dis. 2018 Oct 15;12(10):e0006882. doi: 10.1371/journal.pntd.0006882 (PMC6201951; doi:10.1371/journal.pntd.0006882)
Supplement: S6 Table — (DOCX) [file pntd.0006882.s006.docx]

| Season/Location | Indoor | | Outdoor | |
| --- | --- | --- | --- | --- |
| Dry | 20 | | 26 | |
| Wet | 21 | | 60 | |
|  |  | |  | |
| Season/Ownership | Public | | Private | |
| Dry | 18 | | 28 | |
| Wet | 34 | | 47 | |
|  |  | |  | |
| Season/Function | Water storage | Discarded item | | Other water receptacle |
| Dry | 14 | 15 | | 17 |
| Wet | 21 | 43 | | 17 |
|  |  | |  | |
| Location/Ownership | Public | | Private | |
| Indoor | 4 | | 37 | |
| Outdoor | 48 | | 38 | |
|  |  | |  | |
| Location/Function | Water storage | Discarded item | | Other water receptacle |
| Indoor | 25 | 3 | | 13 |
| Outdoor | 10 | 55 | | 21 |
|  |  | |  | |
| Ownership/Function | Water storage | Discarded item | | Other water receptacle |
| Public | 8 | 28 | | 16 |
| Private | 27 | 30 | | 18 |

**S6 Table.** The number of containers positive for juvenile *Ae. aegypti* by characteristics.
