# Supplementary material for: Location, seasonal, and functional characteristics of water holding containers with juvenile and pupal Aedes aegypti in Southern Taiwan: A cross-sectional study using hurdle model analyses
Source: PLoS Negl Trop Dis. 2018 Oct 15;12(10):e0006882. doi: 10.1371/journal.pntd.0006882 (PMC6201951; doi:10.1371/journal.pntd.0006882)
Supplement: S7 Table — (DOCX) [file pntd.0006882.s007.docx]

| **S7 Table.** Zero-inflated model vs. zero-truncated | | | |  |  |  |  |  |
| --- | --- | --- | --- | --- | --- | --- | --- | --- |
| Stage | Juvenile |  |  |  | Pupae |  |  |  |
| Method | zero-inflated |  | zero-truncated* |  | zero-inflated |  | zero-truncated* |  |
| Distribution | Poisson | NB** | Poisson | NB** | Poisson | NB** | Poisson | NB** |
| Predictors | Season  Ownership  Location  Function  Season: Function  Ownership: Function  Location: Function | Season  Ownership | Season  Ownership  Location  Function  Season: Function  Ownership: Function  Location: Function | Season  Ownership | Season  Ownership  Location  Function  Season: Ownership  Season: Function  Ownership: Location  Location: Function | Season  Location  Function  Location: Function | Season  Ownership  Location  Function  Season: Function  Ownership: Location  Location: Function | Season  Location  Function  Location: Function |
| AIC | 7276.94 | 1594.41 | 7276.94 | 1594.50 | 625.74 | 522.92 | 626.76 | 521.89 |
| * Hurdle |  |  |  |  |  |  |  |  |
| ** Negative binomial | |  |  |  |  |  |  |  |
